# Multilevel analysis of intimate partner violence and associated factors among pregnant women in East Africa: Evidence from recent (2012–2018) demographic and health surveys

**DOI:** 10.1186/s13690-023-01065-8

**Published:** 2023-04-23

**Authors:** Desale Bihonegn Asmamaw, Wubshet Debebe Negash, Desalegn Anmut Bitew, Tadele Biresaw Belachew

**Affiliations:** 1 Department of Reproductive Health, Institute of Public Health, College of Medicine and Health Sciences, University of Gondar, Gondar, Ethiopia; 2 Department of Health Systems and Policy, Institute of Public Health, College of Medicine and Health Sciences, University of Gondar, Gondar, Ethiopia

**Keywords:** Intimate partner violence, Pregnant women, DHS, East Africa

## Abstract

**Background:**

Globally, intimate partner violence (IPV) during pregnancy is the most common and major public health problem. It has a negative effect on the lives of both mother and fetus. Despite its prominence, many countries in East Africa have paid little attention to this issue. This study assessed the prevalence and associated factors of intimate partner violence among pregnant women in East African countries.

**Methods:**

The study adopted a secondary method data analysis that utilized recent Demographic and Health Surveys of 10 countries in East Africa between 2012 and 2018. A total of 23,521 women who gave birth in the 5 years preceding the survey were included. A multilevel mixed-effect logistic regression model was fitted to identify factors associated with IPV. Variables with a p-value < 0.05 were declared as significant factors associated with IPV.

**Results:**

The overall prevalence of IPV in East Africa was 37.14 (95% CI 36.53, 37.76). Women with age 25–34 (AOR = 1.20;95%CI; 1.06, 1.36), 35–39 (AOR = 1.40;95%CI; 1.24, 1.58), and 40–49 (AOR = 1.66;95%CI; 1.43, 1.95), women with no education (AOR = 1.27;95%CI; 1.16, 1.39), women with no occupation (AOR = 1.36; 95%CI; 1.27, 1.47), women from households with the poorest (AOR = 1.51; 95%CI: 1.33, 1.71), poorer (AOR = 1.40;95% CI:1.24, 1.58), middle (AOR = 1.32;95%CI:1.17, 1.48), and richer (AOR = 1.26;95%CI: 1.13, 1.40), husband drinks alcohol (AOR = 2.54; 95%CI 2.39, 2.71), ≥ 5 number of living children (AOR = 1.28; 95%CI: 1.31, 2.57) and rural areas (AOR = 1.14; 95%CI: 1.03, 1.25) were significantly associated with IPV.

**Conclusion:**

More than one-third of pregnant women experienced intimate partner violence in East Africa. Promoting the educational status of women, the economic capacity of women, and the healthy behavior of the husband by reducing alcohol consumption, with particular attention to rural women and violence during pregnancy, is vital to reduce the prevalence of IPV.

**Supplementary Information:**

The online version contains supplementary material available at 10.1186/s13690-023-01065-8.

## Introduction

Intimate partner violence (IPV) is defined as abuse or aggression in a romantic relationship that causes sexual, physical, and psychological harm to those involved [1]. It is one of the most common types of gender-based violence. IPV includes physical, sexual, and emotional abuse and controlling behaviors by an intimate partner [2].

IPV is a common public health issue and human rights violation against pregnant women [3]. Some risk factors may become even more significant during pregnancy, resulting in violence or aggravating it, since pregnancy can require more relationship commitment and resources [4]. Approximately more than 324,000 women per year experience IPV during pregnancy [5]. According to the WHO report, the global prevalence of IPV during pregnancy was 38%, with the highest prevalence accounted in Africa (33%) [6]. Furthermore, the overall IPV during pregnancy is higher in developing countries (27.7%) than in developed countries (13.3%) [7]. The prevalence of IPV among pregnant women was 28.74%, 33%, and 37% in Ethiopia [8], Nigeria [9], and Kenya [10], respectively.

IPV during pregnancy has special concern due to the potential negative impacts on both mothers and their fetuses [11]. It may lead to many complications, such as miscarriage, antepartum hemorrhage, preeclampsia, and gestational diabetes [12, 13]. It also leads to sexually transmitted infections and mental disorders such as eating disorders, sleep disorders, depression, and anxiety [8]. In addition, IPV during pregnancy is linked to high perinatal and neonatal morbidity and mortality [14–16]. Intrauterine growth retardation, low birth weight, and preterm delivery are common perinatal and neonatal complications that happen because of pregnancy-related IPV [8, 17].

Eliminating violence against women and girls is pivotal to achieving gender equality, women’s empowerment, and the Sustainable Development Goals (SDGs). The World Health Organization (WHO) and UN Women, in collaboration with ten other UN, bilateral, and multilateral agencies, have developed “RESPECT Women in order to prevent violence against women [18]. According to research conducted in Kenya, Uganda, and Tanzania, several preventive measures taken in these countries have included screening for IPV in reproductive health programs and antenatal, community awareness campaigns like the Start phase, followed by the Awareness phase, then Support, and finally Action (SASA) intervention programme that used strategies of advocacy, capacity building, community activism, distribution of learning materials, youth and men’s programming; and programmes implemented with an HIV and IPV-integrated approach to inform policy and programming [19, 20]. Models of these programs, such as SASA, have significantly reduced IPV at the community level, but their effectiveness has been questioned at the population level [19, 20].

Although studies on intimate partner violence in Africa are limited, available data showed that 36.6% of women in Africa experienced lifetime IPV among ever-partnered women [21]. About 28.74% of women in Ethiopia [22], 37% of women in Kenya [23], 35.1% of women in Rwanda [24] ,44.6% of women in Nigeria [25] experienced IPV during pregnancy. Scholars revealed that individual characteristic of the women and husbands, and socio cultural factors have been identified as one of the significant factors of IPV. Findings in the literature point to gender based power and socio economic inequality as determinants of IPV and it has serious mental, sexual, and reproductive health problems for the survivors, it has also related with high social and economic cost. Women vulnerability, in terms of lower education, low-income status, unemployment has been identified as contributing factors for IPV [6, 8, 9, 15, 26–28]. Findings showed that less educated women, unemployed, women living in rural areas, and women living in low-income household increase the risk of experiencing IPV [6, 8, 9, 15, 26–28]. Moreover, women with low socio economic status (physical assets, financially), male personality disorders, weak criminal sanctions against perpetrators of GBV or against violence, exposure to violence in childhood were more likely to experience IPV during pregnancy [25, 29].

Even though the prevalence of IPV among pregnant women has significant in worldwide, limited studies are conducted on the prevalence and associated factors of IPV among pregnant women and these studies were limited to non-pregnant women, and all of them focused on specific parts of the country [27]. As to our search of the literature, no study has been conducted to investigate the prevalence and related factors of IPV based on the pooled Demographic and Health Surveys (DHSs) data. Investigating the prevalence of IPV and its associated factors in East Africa countries is crucial to assess cross-national disparities in women’s autonomy. Besides, the study had adequate statistical power to detect the true effects of variables; hence, it is based on the pooled DHS data in East Africa countries. An important benefit of this study is that it will serve as input to program planners, who will use the results to allocate resources for improving maternal and child health. Therefore, the aim of this study is to determine IPV and associated factors in East African countries.

## Methods

The secondary data analysis was conducted based on the most recent 10 East African countries (Burundi, Ethiopia, Kenya, Comoros, Malawi, Rwanda, Tanzania, Zambia, Zimbabwe, and Uganda) demographic health survey (DHS) datasets from 2012 to 2018. These datasets were appended together to investigate intimate partner violence and associated factors among pregnant women in East Africa.

The data were obtained from the DHS program’s official database, which can be found at www.measuredhs.com. DHS is nationally representative household surveys that provide data that is comparable across the countries for monitoring and impact evaluation indicators in the areas of population, health, and nutrition. In each country, the surveys utilized a similar design. The DHS sample was stratified and selected in two stages. Each region was stratified into urban and rural areas. Samples of enumeration areas (EAs) were selected independently in each stratum in two stages. Implicit stratification and proportional allocation were achieved at each of the lower administrative levels by sorting the sampling frame within each sampling stratum before sample selection, according to administrative units in different levels, and by using a probability proportional to size selection at the first stage of sampling. In the first stage, enumeration areas (EAs) or primary sampling units (PSUs) are randomly selected in clusters based on district and rural/urban residence. Enumeration areas are the primary sampling units (PSU) for the area frame.

In the second stage an average of 28 households per each EA were randomly selected. One eligible person was randomly selected from each eligible household to respond to an interview following WHO’s guidelines on the ethical collection of information on IPV. According to DHS procedures, one woman between 15 and 49 years of age was randomly selected for the IPV module in two-thirds of households. For the remaining third of households, a random man between the ages of 15 and 54 was selected for the IPV module [30]. We used DHS surveys done in 10 East African countries and a weighted sample of 23,521 women with children in the five years before the surveys who were selected and interviewed for the intimate partner violence module and had complete cases on all variables of interest from Burundi (3147), Ethiopia (1966), Kenya (1769), Comoros (857), Malawi (2739), Rwanda (915), Tanzania (3157), Uganda (2856), Zambia (3329) and Zimbabwe (2794) were included in the current study to assess whether they had experienced intimate partner violence during their pregnancy.

### Outcome variable

The outcome variable for this study was intimate partner violence (IPV). IPV is defined as any behavior within an intimate relationship that causes physical, emotional, or sexual harm to those in the relationship, whether they are current or former partners. The modified Conflict Tactic Scales of Straus were used to measure the outcome variables [31]. Women were asked whether they had experienced the acts forwarded by their husband/partner for currently married women and recently married women during their pregnancies. Then, the women’s self-reported responses to questions were used to decide the women’s IPV experience [8] (Table 1). Thus, respondents were categorized as having experienced IPV if they reported experiencing at least one act of IPV during pregnancy.

**Table 1:** Question used to asses intimate partner violence

Types of IPV	Questions used to asses violence
Physical violence	Ever been kicked or dragged by your husband?
	Ever been strangled or burned by a husband?
	Ever been threatened with a knife, gun, or another weapon?
Sexual violence	Ever been physically forced to have unwanted sex by your husband?
	Ever been forced to do other sexual acts by your husband?
	Ever been forced to perform sexual acts respondent didn’t want to?
Emotional violence	Ever been humiliated by your husband?
	Ever been threatened with harm by your husband?
	Ever been insulted or made to feel bad by your husband?

### Independent variable

Age of the women (15–24, 25–34, 35–39, and 40–49), women’s education (no formal education, primary education, and secondary education), women’s occupation (working, not working), wealth index (poorest, poorer, middle, richer, and richest), husband education (no formal education, primary education, and secondary and above ), husband drinks alcohol (yes, no), spousal age gap (< 5, ≥ 5), sex of household head (male, female), number of living children (0, 1–2, 3–4, and ≥ 5), and media exposure (yes, no) were considered as individual-level variables.

Community-level variables, which were created by aggregating individual-level data into clusters, included community level poverty, community level education and community level media exposure. From the community level variables, place of residence and countries retained original categorizations. Place of residence is one of the criteria utilized in designing the sample to estimate the prevalence of core demographic and health indicators at the national level. It is categorized as ‘rural’ or ‘urban’ and it directly explains community characteristics. However, community media exposure, community level poverty, and community-level education were constructed by aggregating individual-level characteristics at the cluster level. All community-level variables are categorized as ‘low’ or ‘high’ representing the magnitude of the phenomena being studied at the cluster level. They were categorized as high or low based on the distribution of the proportion values computed for each community after checking the distribution by using the histogram. The aggregate variable was not normally distributed, and the median value was used as a cut-off point for the categorization, We used overall media for all countries [32, 33]. Community-level factors describe groups of populations living in similar settings (EA levels) [34]. Community-level poverty was categorized as low if the proportion of households which is from households belonging to the categories of poor was less than 50% and categorized as high if the proportion was greater than 50%. Community-level media exposure was coded as “0” for low (communities in which < 50% of women had media exposure for at least one media), “1” for high community-level media exposure (communities in which ≥ 50% of women had media exposure for at least one media) [35, 36]. Community-level education was also categorized high or low based on national media value (50% percentiles) [37, 38].

### Data analysis

Stata version 14 statistical software was used for data analysis. All frequency distributions were weighted (v005/1,000,000) throughout the analysis to ensure that the DHS sample was a representative sample and to obtain reliable estimates and standard errors before data analysis.

The first step was a graphical representation of intimate partner violence among pregnant women in East Africa. The second step was the bivariable analysis that calculated the proportion of IPV across the independent variables with their *p*-values. All the variables that were shown to be statistically significant in the bivariable analysis and used for multi-level analysis. In the final step of the analysis, a multilevel logistic regression analysis comprising fixed effects and random effects was done. Multilevel mixed-effects complementary logistic regression models were developed to accommodate the stratified multistage sampling technique used in the DHS. Multilevel mixed-effects models can also be used to assess the effect of hierarchical ordering (PSUs and regions) on the variance of associated factors [39].

The outcome variables were unevenly distributed, so the complementary logistic regression function was used instead of the normal binary logistic regression function. In this study, the dependent variables were binary, but unevenly distributed. As a result, the distribution of outcomes does not meet the symmetrical assumption in a normal binary logistic regression model. In multilevel mixed-effects complementary log regression, the symmetrical assumption of binary logistic regression is relaxed, thereby making it possible to avoid biased parameter estimates when modeling events with asymmetrical distributions [40, 41].

The results of the fixed effects of the model were presented as an adjusted odds ratio (AOR) while the random effects were assessed with Intra-Cluster Correlation (ICC). Taking clusters as a random variable, the ICC reveals the variation of IPV between clusters is calculated as;$ICC=\frac{VA}{VA+3.29}*100\%$ [42–44]. Simultaneously, model fitness was done using the deviance (-2LLR). A two-level multilevel binary logistic regression model was applied on pooled data for all countries surveys to assess the IPV effects of several individual and community level factors on IPV in east Africa countries. Four models were fitted; the null model (Model 0) shows the variations in IPV in the absence of any independent variables. Model I adjusted for the individual-level variables, Model II adjusted for the community-level variables, and Model III adjusted for both individual and community-level variables. All significantly associated factors from the bivariate analyses were included in multilevel analysis (p < 0.05). The fixed effects or measure of association was used to estimate the association between the likelihood of magnitude of IPV and individual and community levels independent variables. It was assessed and the strength was presented using Adjusted Odds Ratio (AOR) and 95% confidence intervals with a p-value of < 0.05.

$$Log \left(\frac{\pi ij}{1-\pi ij}\right)=\beta o+ \beta 1xij+ \beta 2xij+\dots uj+eij$$

Where,$\pi ij$: the probability of IPV, $1-\pi ij$: the probability of not experienced IP. ß0 is intercept that is the effect of IPV when the effect of all independent variables is absent. $\beta 1xij$ are individual and community level variables for the i^th^ individual in group j, respectively. The ß’s are fixed coefficients indicating a unit increase in X can cause a ß unit increase in probability IPV. The uj shows the random effect for the j^th^ clusters [42, 44, 45].

## Results

### Socio-demographic factors

The current study included 23,521 (weighted) married pregnant women. The median age of the women was 29 years old, with an interquartile range of 24 to 35, and about 46.45% of the women were aged 25–34 years old. More than two-thirds (71.67%) of respondents had no occupation. About 50.27 and 45.56% of pregnant women and their husbands had primary education, respectively. Nearly two-fifths (39.96%) of women had a husband who drinks alcohol, and nearly two-thirds (66.44%) of study participants were exposed to media. Regarding the community-level factors, nearly three-fourths of participants (73.13%) were rural dwellers. More than half (53.46%) of pregnant women were from communities with high illiteracy levels (Table 2).

**Table 2:** Individual and community level factors of IPV in East Africa according to recent demographic and health survey from 2012 to 2018

Variables	Weighted frequency	Percentage (%)
**Women age**		
15–24	6,6526	28.27
25–34	10,930	46.45
35–39	3452	14.67
40–49	2495	10.60
**Women education**		
No formal education	4783	20.33
Primary education	11,827	50.27
Secondary education and above	6919	29.40
**Women occupation**		
Working	6659	28.33
Not working	16,843	71.67
**Husband education**		
No formal education	3543	16.80
Primary education	9611	45.56
Secondary education and above	7940	37.64
**Sex of household head**		
Male	17,889	76.02
Female	5641	23.98
**Husband drinks alcohol**		
Yes	9401	39.96
No	14,126	60.04
**Spousal age gap**		
< 5	11,646	56.20
≥ 5	9076	43.80
**Wealth index**		
Poorest	4873	20.71
Poorer	4706	20.00
Middle	4513	19.18
Richer	4837	20.56
Richest	4601	19.55
**Number of living children**		
0	239	1.02
1–2	11,396	48.43
3–4	7186	30.54
≥ 5	4709	20.01
**Media exposure**		
Yes	15,628	66.44
No	7893	33.56
**Resident**		
Rural	17,213	73.13
Urban	6317	26.85
**Community media exposure**		
Lower	12,009	51.04
Higher	11,521	48.96
**Community-level poverty**		
Lower	13,321	56.61
Higher	10,208	43.39
**Community-women education**		
Lower	12,579	53.46
Higher	10,951	46.54

### Prevalence of intimate partner violence

**Fig. 1:**
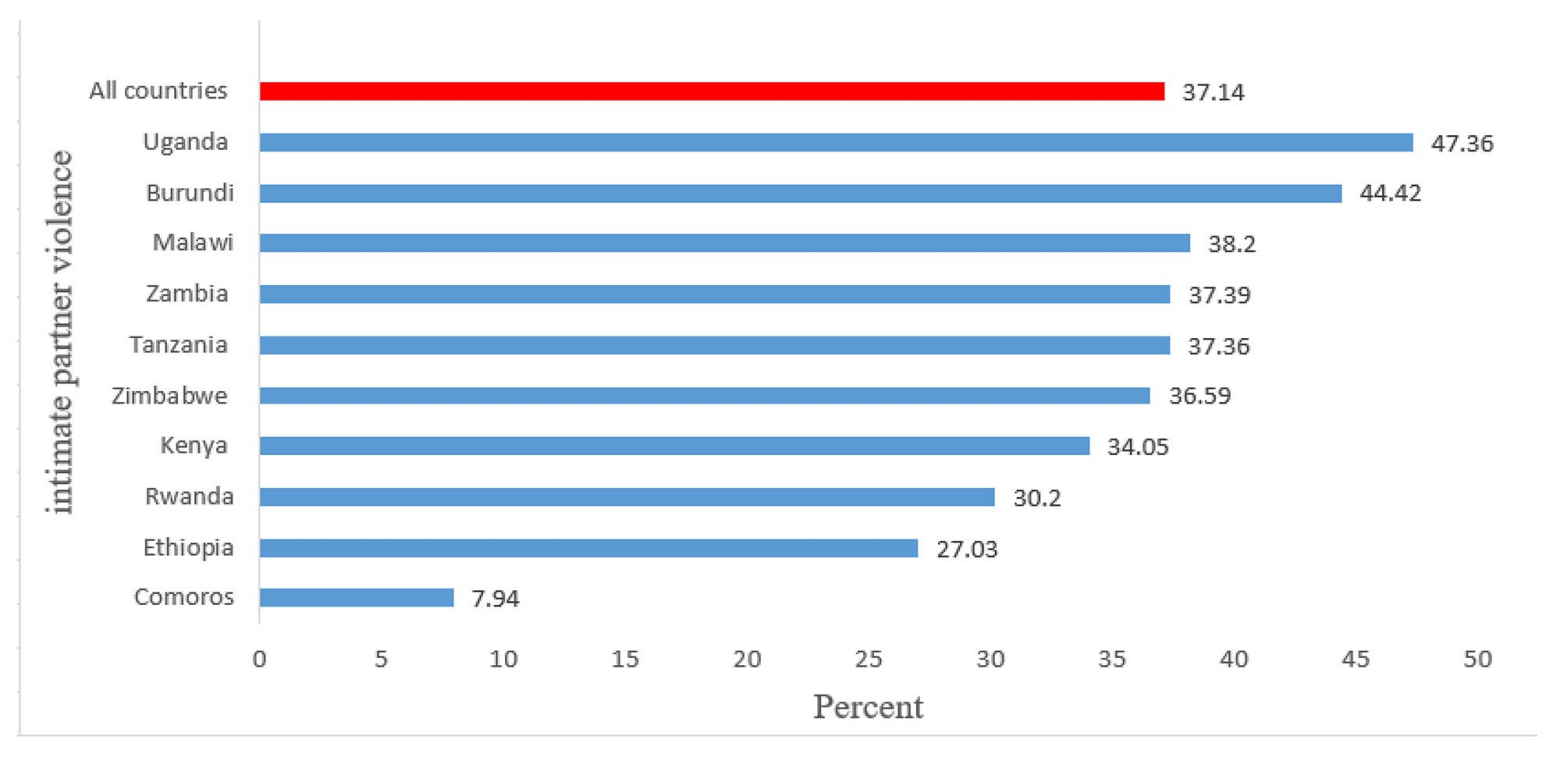
Prevalence of intimate partner violence in East Africa according to recent demographic and health survey from 2012 to 2018 **List of tables**

The overall prevalence of IPV in East Africa countries was 37.14 (95% CI 36.53, 37.76), with the highest prevalence occurred in Uganda (47.36%) and the lowest prevalence occurred in Comoros (7.94%) (Fig. 1).

### Factors associated with intimate partner violence during pregnancy

The Intra-cluster Correlation Coefficient (ICC) and Likelihood ratio (LR) tests were performed. In the null model, the ICC was 0.44 (95% CI 0.34, 0.55), indicating that cluster differences accounted for around 44% of the IPV variation, and individual differences accounted for the remained. In terms of goodness of fit, model 3, which incorporated both individual and community-level factors, was selected to predict the IPV among pregnant women. This model was selected because it has the lowest (25105.1) deviance as compared with the rest of the models.

Regarding the fixed effects, age of the respondent, women’s education, residence, occupation, wealth index, husband drinks alcohol, country, and number of living children were significantly associated with intimate partner violence.

The odds of experiencing IPV among pregnant women with the age groups 25–34, 35–39, and 40–49 was 20% (AOR = 1.20;95%CI; 1.06, 1.36), 40% (AOR = 1.40;95%CI; 1.24, 1.58), and 66% (AOR = 1.66;95%CI; 1.43, 1.95) times higher as compared to women under the age group of 15–24 years, respectively. Besides, pregnant women with no education was 27% (AOR = 1.27;95%CI; 1.16, 1.39) times increased odds of experiencing IPV as compared to those who had higher education. The likelihood of experiencing IPV among pregnant women with no occupation was 36% (AOR = 1.36; 95% CI; 1.27, 1.47) times higher as compared to their counterparts. The odds of experiencing IPV among pregnant women from the poorest, poorer, middle, and richer households was 1.51 (AOR = 1.51; 95% CI: 1.33, 1.71), 1.40 (AOR = 1.40; 95% CI: 1.24, 1.58), 1.32 (AOR = 1.32; 95% CI:1.17, 1.48), and 1.26 (AOR = 1.26; 95% CI: 1.13, 1.40) times higher as compared to those from households with richest wealth quantile, respectively. Pregnant women whose husband drinks alcohol was 2.54 (AOR = 2.54; 95% CI 2.39, 2.71) times increased odds of experiencing IPV as compared to women who had a husband with no drink alcohol. Regarding number of living children, the likelihood of experiencing IPV among pregnant women with ≥ 5 number of living children was 1.84 (AOR = 1.84; 95%CI: 1.31, 2.57) and 1.78 times higher as compared to their counterparts. Pregnant women from rural areas had 14% (AOR = 1.14; 95% CI: 1.03, 1.25) increased odds of experiencing IPV as compared to urban dwellers (Table 3).

**Table 3:** Multivariable multilevel logistic regression model results of IPV in East Africa countries using recent demographic and health survey from 2012 to 2018

Variables	Null model	Model I (AOR, 95%CI)	Model II (AOR, 95%CI)	Model III (AOR, 95%CI)
Age of the respondent				
15–24		1		**1**
25–34		1.21 (1.06, 1.37)		**1.20 (1.06, 1.36)**
35–39		1.41 (1.25, 1.59)		**1.40 (1.24, 1.58)**
40–49		1.66 (1.43, 1.92)		**1.66 (1.43, 1.95)**
Women education				
No formal education		1.27 (1.16, 1.39		**1.27 (1.16,1.39)**
Primary education		1.08 (0.97, 1.22)		1.07 (0.95, 1.20)
Secondary education and above		1		1
Women occupation				
Working				1
Not working		1.355027 (1.26, 1.45)		**1.36 (1.27, 1.47)**
Husband education				
No formal education		1.10 (0.98, 1.21)		1.10 (0.97, 1.21)
Primary education		1.02 (0.91, 1.13)		1.03 (0.91, 1.13)
Secondary education and above		1		
Husband drinks alcohol				
Yes		2.54 (2.39, 2.71)		**2.54 (2.39, 2.71)**
No		1		1
Spousal age gap				
< 5		1.02 (0.95, 1.08)		1.02 (0.96, 1.08)
≥ 5		1		
Sex of household head				
Male		1.10 (1.01, 1.20)		1.10 (0.97, 1.17)
Female		1		1
Wealth index				
Poorest		1.43 (1.28, 1.61)		**1.51 (1.33, 1.71)**
Poorer		1.33 (1.19, 1.48)		**1.40 (1.24, 1.58)**
Middle		1.24 (1.12, 1.38		**1.32 (1.17, 1.48)**
Richer		1.22 (1.11, 1.36)		**1.26 (1.13, 1.40)**
Richest				1
Number of living children				
0		1		1
1–2		0.95 (0.70, 1.32)		0.96 (0.69, 1.32)
3–4		1.33 (0.96, 1.85)		1.33 (0.96, 1.85)
≥ 5		1.84 (1.31, 2.57)		**1.84 (1.31, 2.57)**
Media exposure				
Yes				1
No				1.04 (0.96, 1.13)
Resident				
Rural			1.17 (1.10, 1.25)	**1.14 (1.03, 1.25)**
Urban			1	1
Community media exposure				
Lower			1	1
Higher			1.03 (0.94, 1.12)	1.03 (0.94, 1.13)
Community-level poverty				
Lower			1.16 (1.07, 1.26)	1
Higher			1	1.08 (0.98, 1.19)
Community-women education				
Lower			0.98 (0.91, 1.07)	1.04 (0.95, 1.14)
Higher			1	1
Country				
Burundi			4.60 (2.21, 6.17)	**3.12 (2.11, 3.96)**
Ethiopia			1.71 (0.96, 2.91)	1.26 (0.91, 1.87)
Kenya			2.78 (1.39, 3.58)	1.67 (0.89, 2.32)
Mali			3.54 (2.02, 4.52)	**2.65 (1.93, 3.97)**
Rwanda			2.11 (1.49, 3.24)	1.41 (0.97, 2.16)
Tanzania			3.59 (2.08, 4.59)	**2.43 (1.71, 3.91)**
Uganda			4.12 (2.76, 5.59)	**2.87 (1.92, 4.12)**
Zambia			2.85 (1.27, 3.36)	**1.89 (1.12, 2.79)**
Zimbabwe			2.48 (1.98, 3.45)	1.39 (0.92, 2.18)
Comoros			1	1
Model Comparison and random effect				
ICC	0.44 (0.34, 0.55)	0.41 (0.32, 0.54)	0.43 (0.34, 0.55)	0.39 (0.31,0.47)
Log-likelihood	-15412.85	-12557.38	-15392.07	-12552.55
Deviance	30825.70	25114.76	30784.14	25105.1

Statistically significant at p-value < 0.05, AOR: Adjusted Odds Ratio, Null

Model: adjusted for individual-level characteristics, Model 2: adjusted for community-level

Characteristics, Model 3: adjusted for both individual and community-level characteristics

Pregnant women from Burundi, Mali, Tanzania, Uganda, Zambia were 3.12 (AOR = 3.12; 95% CI: 2.11, 3.96), 2.65 (AOR = 2.65; 95% CI: 1.93, 3.97), 2.43 (AOR = 2.43; 95% CI: 1.71, 3.91), 2.87 (AOR = 2.87; 95% CI: 1.92, 4.12), and 1.89 (AOR = 1.89; 95% CI: 1.12, 2.79) times higher as compared to those from Comoros.

## Discussion

This study aimed to assess the prevalence and associated factors of intimate partner violence among pregnant women in East Africa. The prevalence of IPV in East African countries was 37.14 (95% CI 36.53, 37.76), with the highest prevalence occurred in Uganda (47.36%) and the lowest prevalence occurred in Comoros (7.94%). Women age, women education, women occupation, wealth index, number of living children, husband drinks alcohol, country, and place of residence were factors significantly associated with IPV.

The prevalence of IPV in this study was in line with a study conducted in Kenya 37% [10], but lower than studies done in Portugal 43.4% [46] and Ethiopia 44.5% [47]. On the other hand the current finding was higher than the previous studies conducted in Ethiopia [8, 28, 48], Nigeria 33% [9], and East Africa 32.6% [15]. The possible explanation for the observed differences could be that the previous studies conducted in Ethiopia [28, 48] and Nigeria [9] were small scale surveys compared with the DHS, which is a nationally representative data survey and covered women in the country. It might be due to the difference in the study population, cultural differences, sample size difference they used, background characteristic among respondents, and implementation of laws that prevent IPV [49, 50]. For example, assessing the magnitude of intimate partner violence (IPV) and its determinant factors among ever married women in Aksum, Ethiopia [51] and East African countries [15]. Moreover, the questions used to assess intimate partner violence are culturally sensitive, hence, the participants might not respond honestly. This could lead to underreporting of the IPV [52].

The current study showed the higher odds of IPV among pregnant women with advanced maternal age as compared to pregnant women of young age. which is supported by the findings in Ethiopia [8], Nigeria [53], and South Africa [16]. The possible reason might be that older women could be more likely to report IPV. Besides, younger women in developing countries, including East Africa are often expected to be passive, quiet, disciplined, shy, and loyal to their partners, so they may not have a probability of reporting IPV [54].

Pregnant women with no formal education had nearly a 27% increase in odds of experiencing IPV as compared to those with secondary and higher education. This result is in line with previous studies done in Ethiopia [8, 11]. The possible justification might be that uneducated pregnant women may have less autonomy to discuss with their husbands to minimize any household disputes. Scholars suggest that education is one way to develop a sense of self-esteem and empower women [8]. Similarly, the number of living children is a significant predictor of IPV during pregnancy. Pregnant women with ≥ 5 living children had higher odds of experiencing IPV as compared to those without children. This finding is consistent with a study conducted in Zimbabwe [17].

Pregnant women whose husband drinks alcohol had a 2.5 times increased odds of experiencing IPV as compared to women who had a non-alcohol drinking husband. This finding is supported by different studies [8, 15, 55]. The possible justification could be that alcohol has direct effects on human physical and cognitive function, reducing self-control and leaving individuals less capable of negotiating a non-violent resolution to conflict within relationships [26, 55]. Moreover, alcohol use is associated with having multiple sexual partners, an issue that may also result in conflict [26]. Women who are from Burundi, Mali, Tanzania, Uganda, Zambia are more likely to experience IPV as compared to those who are form Comoros. The possible justification might be due to the difference in sociodemographic characteristics and cultural variations.

Furthermore, residence was an important factor in IPV. Pregnant women from rural areas had higher odds of experiencing IPV as compared to urban areas. This finding is supported by the previous study done in Ethiopia [8]. Women in rural areas are not autonomous, educated, or informed about gender equality. As a result, they could have been embarrassed by decision-making in the household [56].

The study’s main strength was that it used nationally representative DHS from recent 10 East African countries, and therefore findings from the sub-region could be generalized. In addition, the DHS uses validated instruments in its appraisals of datasets, along with its large sample size and well-designed procedures, such as training field enumerators and employing well-tested methods for data collection. Even if important findings were found in the current study, the cross-sectional nature of the study did not show a cause-and-effect relationship between the outcome and the explanatory variables. Since DHS data did not include qualitative data, we are unable to address the association of qualitative variables such as attitudes and perceptions of pregnant women and society towards IPV.

## Conclusion

More than one-third of pregnant women experienced intimate partner violence in East Africa. IPV during pregnancy was significantly associated with women’s age, education, wealth index, number of living children, husband drinks alcohol, country, and residence. To reduce the prevalence of IPV, it is critical to promote women’s education, economic capacity, and husband’s healthy behavior by reducing alcohol consumption, with a special focus on rural women and violence during pregnancy. The government, in collaboration with non-governmental organizations (NGO), should provide training on IPV for health care providers to screen and provide holistic care to violence victims. Furthermore, qualitative research is recommended to assess the attitudes and perceptions of pregnant women toward IPV.

## Electronic supplementary material

Below is the link to the electronic supplementary material.

Supplementary Material 1

Supplementary Material 2

## Data Availability

This study used data from the most recent Demographic and Health Survey, which is freely available online at (https://www.dhsprogram.com).
