# Supplementary material for: Multilevel analysis of intimate partner violence and associated factors among pregnant women in East Africa: Evidence from recent (2012–2018) demographic and health surveys
Source: Arch Public Health. 2023 Apr 23;81:67. doi: 10.1186/s13690-023-01065-8 (PMC10122807; doi:10.1186/s13690-023-01065-8)
Supplement: Supplementary file 1 — Supplementary Material 1 [file 13690_2023_1065_MOESM1_ESM.docx]

**Letter to Editor**

Violence against women is largely recognized as a major human rights abuse and a significant public health problem for both the mother and the fetus she bears [1]. It is one of the barriers to achieving sustainable development goals due to its adverse health consequences. Studies demonstrate that violence during pregnancy is more common in developing countries (as high as 32%) than in developed countries (less than 12%) [2]. The consequences of intimate partner violence (IPV) during pregnancy range from financial hardships to high rates of maternal and neonatal morbidity and mortality. Moreover, pregnant women who have experienced violence are also likely to delay the use of maternal health care services like antenatal care, place of delivery, and postnatal care [3].

Even though the prevalence of IPV is significant worldwide and some studies are conducted on the prevalence of IPV, most of these studies were limited to non-pregnant women, and almost all of them focused on specific parts of the country [2-5]. The current study used multilevel analysis to model the hierarchical nature of the data, which differed from the previous studies. Community-level variables such as poverty, media exposure, and education were not included in previous studies; however, these variables were included in this study. Moreover, the current study tried to assess additional factors such as the spousal age gap, number of living children, media exposure, and sex of the household head. Evidences revealed that all the aforementioned factors were important to determine IPV [5, 6].

As to our search of the literature, no study has been conducted to investigate the prevalence and related factors of IPV among pregnant women in East African countries based on the pooled Demographic and Health Survey (DHS) data. Investigating the prevalence of IPV and its associated factors in East African countries is crucial to assessing cross-national disparities in women's autonomy. Besides, the study had adequate statistical power to detect the true effects of variables; hence, it is based on the pooled DHS data in East African countries. An important benefit of this study is that it will serve as input to program planners, who will use the results to allocate resources for improving maternal and child health. Therefore, the aim of this study is to determine IPV and associated factors among pregnant women in East African countries.

This finding revealed more than one-third of pregnant women experienced intimate partner violence in East Africa. Which implies thousands of pregnant women suffer from major public health problems like multiple adverse physical, mental, sexual, and reproductive health effects. In addition, it has increase the adverse birth outcome like preterm birth, small for gestational age (SGA), low birth weight (LBW), stillbirths, and miscarriage. This raise the risk of maternal and child mortality. Therefore, it is crucial to counteract the problem by working on preventing IPV during pregnancy.

References

1. Runyan D, Wattam C, Ikeda R, Hassan F, Ramiro L: Child Abuse and Neglect by Parents and Other Caregivers (From World Report on Violence and Health, P 57-86, 2002, Etienne G. Krug, Linda L. Dahlberg, et al., eds.–See NCJ-197425). *Geneva: World Health Organization* 2002.

2. Yimer T, Gobena T, Egata G, Mellie H: Magnitude of domestic violence and associated factors among pregnant women in Hulet Ejju Enessie District, Northwest Ethiopia. *Advances in public health* 2014, 2014.

3. Musa A, Chojenta C, Geleto A, Loxton D: The associations between intimate partner violence and maternal health care service utilization: a systematic review and meta-analysis. *BMC women's health* 2019, 19(1):1-14.

4. Devries KM, Kishor S, Johnson H, Stöckl H, Bacchus LJ, Garcia-Moreno C, Watts C: Intimate partner violence during pregnancy: analysis of prevalence data from 19 countries. *Reproductive health matters* 2010, 18(36):158-170.

5. Shitu S, Yeshaneh A, Abebe H: Intimate partner violence and associated factors among reproductive age women during COVID-19 pandemic in Southern Ethiopia, 2020. *Reproductive health* 2021, 18(1):1-10.

6. Chernet AG, Cherie KT: Prevalence of intimate partner violence against women and associated factors in Ethiopia. *BMC women's health* 2020, 20(1):1-7.
